# Association between mild depressive states in polycystic ovary syndrome and an unhealthy lifestyle

**DOI:** 10.3389/fpubh.2024.1361962

**Published:** 2024-04-12

**Authors:** Lingling Li, Zhiyuan Kang, Ping Chen, Baihan Niu, Yaohui Wang, Liping Yang

**Affiliations:** ^1^Department of Obstetrics and Gynecology, The First Affiliated Hospital of Henan University of Chinese Medicine, Zhengzhou, Henan Province, China; ^2^First Clinical Medical College of Henan University of Chinese Medicine, Zhengzhou, Henan Province, China; ^3^Henan University of Chinese Medicine, Zhengzhou, Henan Province, China

**Keywords:** polycystic ovary syndrome, depressive symptoms, high-fat diet, staying up late, lack of exercise

## Abstract

**Objective:**

Polycystic Ovary Syndrome (PCOS) is a prevalent and frequently encountered gynecological disorder. Its high variability and the complexities associated with its management often lead to psychological stress in affected women, manifesting in symptoms of depression. Embracing a healthy lifestyle is fundamental in PCOS treatment. Consistent adherence to a healthy lifestyle not only aids in improving PCOS symptoms but also plays a role in enhancing mental well-being. However, there is currently limited research examining the extent of depression, its prevalence, and its correlation with lifestyle among individuals with PCOS. Therefore, this study aims to explore the impact of lifestyle factors on the depressive state of individuals with PCOS.

**Methods:**

This cross-sectional study gathered data from 411 individuals with PCOS at a comprehensive hospital in Henan, China. Depression status was assessed using the Hamilton Depression Scale, and demographic information as well as lifestyle habits were simultaneously collected. Univariate and multivariate analyses using logistic regression were conducted to identify risk factors associated with the depressive state in PCOS.

**Results:**

Among the surveyed 411 individuals with PCOS, approximately 49.4% exhibited symptoms of depression, with 83.7% experiencing mild depressive symptoms. A disease duration of 1–3 years, the presence of acne, and unhealthy lifestyle factors such as high-fat diet, staying up late, lack of exercise, and mental stress emerged as significant risk factors for the onset of depressive symptoms.

**Conclusion:**

Depressive symptoms in individuals with PCOS are predominantly mild. The risk of comorbid depression in PCOS is associated with the presence of acne, frequent high-fat diet, regular staying up late, lack of exercise, and mental stress.

## 1 Introduction

Polycystic Ovary Syndrome (PCOS) affects 5–21% of women and is a disorder characterized by reproductive dysfunction, metabolic disturbances, and psychological abnormalities. It exerts a significant impact on the entire lifespan of affected women (1). Compared to healthy women, individuals with PCOS experience a higher incidence of psychological issues and exhibit poorer self-recovery capabilities (2). The latest literature analysis indicates that the clinical prevalence of depression in women with PCOS is around 37% or even higher, surpassing that of healthy individuals by more than four times (3). Postpartum depression (4, 5) and the risk of neurodevelopmental and psychiatric disorders in offspring (6) are also significantly elevated in individuals with PCOS. PCOS, when accompanied by depression, persists throughout a woman’s life and proves challenging to cure, imposing a substantial economic and psychological burden on both society and individuals. Therefore, current guidelines both domestically and internationally highlight the importance of conducting depression screening for individuals with PCOS at their initial medical consultations (7), underscoring the growing recognition of the psychological issues associated with this condition. However, there is no standardized treatment protocol, which may be attributed to the lack of clarity in reported levels of depression. Lifestyle adjustments and psychological counseling may contribute to the improvement of mild depressive states in individuals with PCOS, preventing further deterioration. For severe depression, intervention with antidepressant medications may be necessary.

The prevalence of PCOS accompanied by depressive symptoms varies across different countries and regions. The occurrence rate of depression in individuals with PCOS ranges from 37 to 52% (8). In conflict-ridden regions such as Syria and Jordan, the prevalence of depression in women with PCOS is reported to be over 65% or even higher (9). In a cross-sectional survey conducted in Pakistan, 80% of PCOS women screened positive for depression (10). Research in China has found varying rates of depression in individuals with PCOS, with an overall prevalence of 39% (11). In the North China region, the rate is reported to be 38% (12), while in Chengdu, the prevalence reaches as high as 51.8% (13). It is evident that the psychological well-being of individuals with PCOS is a subject of concern. However, the lack of specific descriptions regarding the severity of depression poses a significant challenge for the implementation of subsequent intervention methods. Early identification of depressive symptoms in individuals with PCOS and timely intervention can effectively prevent the onset of depression in these individuals.

PCOS is a highly heterogeneous disorder encompassing reproductive dysfunction, metabolic disturbances, and various clinical manifestations. Current treatments primarily focus on symptomatic relief, with a cornerstone being a healthy lifestyle, which proves to be the most fundamental and effective intervention. It not only aids in improving the psychological state but also becomes challenging to maintain due to the involvement of depression. This not only exacerbates clinical symptoms of PCOS but also intensifies the psychological burden, creating a vicious cycle between the two. Recent research has revealed a significant increase in the risk of binge eating among individuals with PCOS (14). Binge eating exacerbates metabolic disruptions in PCOS, leading to elevated levels of inflammatory factors, disturbances in gut microbiota, and triggering neuroinflammation. Consequently, this worsens the phenotypic expression of PCOS and increases the risk of depression. Prolonged periods of sedentary behavior and lack of physical activity contribute to weight gain and metabolic abnormalities in patients. Therefore, an unhealthy lifestyle may indeed heighten the risk of both PCOS and its associated depressive symptoms.

The First Affiliated Hospital of Henan University of Chinese Medicine is a tertiary comprehensive hospital in Henan, China. With an annual outpatient volume of up to 100,000 in the Obstetrics and Gynecology department, to some extent, it can reflect the prevalence of PCOS accompanied by depression in the local area. This study aims to investigate the prevalence and severity of depression in individuals with PCOS, exploring the relationship between lifestyle, general conditions, and depressive symptoms. A comprehensive understanding of the relationships between research factors contributes to the development of effective intervention strategies. The goal is to enhance the lifestyle of individuals with PCOS, reduce depressive states, and mitigate the occurrence of depression, ultimately improving the quality of life for individuals with PCOS.

## 2 Materials and methods

### 2.1 Study design

From January 2023 to June 2023, this cross-sectional study was conducted at the First Affiliated Hospital of Henan University of Chinese Medicine. The study utilized a simple random sampling method, focusing on individuals with PCOS attending the Obstetrics and Gynecology outpatient clinic as the research subjects.

### 2.2 Sample size calculation

Using the formula for calculating the overall rate in a cross-sectional survey.


$$n={\left(\frac{Z_{1-\alpha /2}}{\delta }\right)}^{2}\times p\times \left(1-p\right)$$

under 95% confidence level (thus Z_1-α/2_ = 1.96), we set *p* = 0.5 (the most conservative method), error rate δ = 5%, and get *N* = 384,15% contingency was taken into account to increase sample size accuracy due to potential non- responses and missing data. The study ultimately completed surveys with 450 cases, ensuring the reliability of the investigation results.

### 2.3 Data collection

This study examined the basic demographic characteristics of the participants, including age, acne, hirsutism, family medical history, and lifestyle factors such as high-fat diet, exercise habits, and bedtime. Data were collected from patients visiting the outpatient department of the First Affiliated Hospital of Henan University of Chinese Medicine who met the diagnostic criteria for PCOS. Information was obtained through the administration of a Chinese version of a paper questionnaire and interviews.

PCOS was diagnosed using the 2018 International Evidence-based Guideline criteria (7), which built on the consensus based 2003 Rotterdam criteria (15). This requires the presence of two of the following: (i) clinical/biochemical hyperandrogenism; (ii) ovulatory dysfunction; and (iii) polycystic ovaries on ultrasound. Exclusion of other etiologies.

The Hamilton Depression Scale-17 was employed to evaluate the depression status of individuals with PCOS (16). A score of ≤7 was interpreted as indicative of individuals with PCOS without depression, whereas a score exceeding 7 signified the presence of individuals with PCOS with depression. Based on the total score, the severity of depression can be classified as follows: Mild: 8-17,Moderate: 18-24,Severe: >24.

Considering that the prevalence of smoking and alcohol abuse among Chinese women is relatively low, lifestyle factors primarily focused on in the assessment include high-fat diet consumption, exercise habits, and late-night activities. A high-fat diet is characterized by a dietary pattern where a minimum of 40–45% of the total daily calories are derived from fat. Additionally, as per the Chinese Dietary Guidelines (17), adults are advised to limit their daily intake of cooking oil to 25–30 grams and trans-fatty acids to 2 grams or less. Common sources of high-fat foods encompass snacks, ice cream, animal fats, dark chocolate, butter, and fried foods. The survey questions focus on the frequency of consuming takeout, fried foods, cakes, ice cream, and snacks, specifically querying whether they are consumed more than three times per week, with responses being either yes or no.

Exercise recommendations for individuals with PCOS are derived from the “WHO Guidelines on Physical Activity and Sedentary Behavior 2020” (18) and the “Recommendations from the 2023 International Evidence-based Guideline for the Assessment and Management of Polycystic Ovary Syndrome” (19). It is suggested that individuals aim for 150–300 min of moderate-intensity exercise or 75–150 min of high-intensity aerobic exercise weekly, including activities like brisk walking, jogging, or cycling. Achieving this goal typically entails exercising for 40 min to 1 h per day, with sessions occurring more than 3 times per week. The survey includes questions regarding whether participants engage in a daily exercise routine of 1 h and whether they exercise more than 3 times a week, with responses being either yes or no.

Among humans, social factors are one of the most significant determinants of sleep duration. Due to the strong demand for social activities, ubiquitous artificial lighting, and the development of communication channels, sleep is often not solely determined by the physiological regulation of the sleep/wake cycle (20). Normally, the optimal time for sleep is between 10 pm and 6 am. Based on the characteristics of social jet lag and chronotype among the Chinese population, with morning types being the majority (21), we define staying up late as going to bed after 11 pm. We inquire about whether individuals go to bed after 11 pm and how often they exceed this time. If it occurs more than 3 times a week, it is considered as “yes”; otherwise, it is “no.”

Perceived Stress refers to the cognitive process in which individuals perceive stress from external or internal stimuli. When individuals feel demands, challenges, or stress from the environment or events, they experience perceived stress. This process is subjective, perceptual, and relatively stable, with different individuals perceiving varying degrees of stress from the same event (22). Therefore, we utilize the Chinese Perceived Stress Scale (CPSS) to assess individual stress levels. Individuals rate the frequency of experiencing each item applicable to them over the past month, on a scale from 0 to 4. The total score ranges from 0 to 40. Based on the reference literature (23), we set stress levels between 0 and 13 as no stress, and above 13 as experiencing stress.

Currently, there is no standardized acne scale. We refer to the questionnaire from the literature (24) to determine the presence or absence of acne. We ask the following question: “Do you (or did you) have acne?” Your acne was diagnosed by: A dermatologist/Your general practitioner/Another physician or surgeon/Another healthcare professional (pharmacist, nurse, physiotherapist, midwife, naturopath, homeopathic practitioner, etc.)/Yourself (self-diagnosis). The response options are yes and no.

Hirsutism is assessed using the modified Ferriman-Gallwey (mFG) scoring system, where a score of ≥8 indicates hirsutism, while a score below 8 indicates the absence of hirsutism (25).

Social demographic characteristics include age (<20, 20–35, and > 35), education level (<9, 9–12, >12 years), duration of illness (<1 year, 1–3 years, >3 years), fertility requirements (yes, no), maternal or sibling history of PCOS (yes, no), and family history of mental illness (yes, no). BMI is calculated using participants’ height and weight, which are collected by trained researchers using standard anthropometric measurements.

Prior to the commencement of the survey, a review of the questionnaire was conducted. All personnel involved in the survey received training to ensure consistency. The HAMD scale requires the patient to be jointly assessed by two trained evaluators. This assessment usually involves conversation and observation. Following the assessment, the two evaluators independently assign scores to the patient. All participants anonymously filled out the questionnaire with their informed consent. This study received approval from the Ethics Review Committee of the First Affiliated Hospital of Henan University of Chinese Medicine (Approval No: 2023HL-200).

### 2.4 Statistical analysis

The demographic details and clinical features of individuals with PCOS exhibiting depressive symptoms were outlined using frequency and percentage. The prevalence of depressive symptoms across various subgroups was presented based on their distinct characteristics. Univariate analysis was conducted using a chi-square test. The comparative variables included age, BMI, fertility demand, acne, hirsutism, family history, high-fat diet habits, exercise habits, frequency of staying up late, and mental stress exposure. Depressive symptoms served as the dependent variable, while statistically significant indicators from univariate analysis were considered independent variables. A binary logistic stepwise backward regression approach was utilized to identify potential risk factors. The multifactor regression model employed in this study primarily examined lifestyle factors such as frequent high-fat diet, infrequent exercise, regular staying up late, and mental stress. Statistical analysis was conducted using IBM SPSS Statistics Version 26.0 (IBM SPSS Inc., Chicago, IL, United States). A significance level of *p* < 0.05 was applied to all analyses. Graphical presentation was prepared using GraphPad Prism software (version 9.0).

## 3 Results

### 3.1 Characteristics of the study subjects

A total of 450 individuals with PCOS were initially included in this study, with 411 individuals (91.3%) considered for further analysis. Among the surveyed patients, 68 (16.1%) were in the adolescent PCOS group, and 345 (83.9%) were in the reproductive-age PCOS group. The majority of participants were aged between 20 and 35, approximately 325 individuals (79.0%). Among them, 255 expressed a desire for fertility, accounting for 62.0%. There were 183 participants classified as obese, representing 44.5%, and 145 individuals with acne, constituting 35.3%. Additionally, 225 participants had dense hair growth, making up 54.7%. Furthermore, 28 individuals (4%) reported a family history of PCOS in their mothers or sisters. In terms of lifestyle, 190 participants (46.2%) reported a high-fat diet, 266 (64.7%) frequently stayed up late, 262 (63.7%) engaged in infrequent physical activity, and 356 (86.6%) experienced chronic stress (Table 1).

**Table 1:** Basic characteristics of survey subjects.

Variable	Total number of people	Percentage (%)	Variable	Total number of people	Percentage (%)
Age (year)			High-fat diet (>3x/week)		
<20	68	16.5	YES	190	46.2
20 ~ 35	325	79.1	NO	221	53.8
>35	18	4.4	Stay up late (>3x/week)		
BMI			YES	266	64.7
<18.5	44	10.7	NO	145	35.3
18.5 ~ 23.9	182	44.3	Frequency of exercise (week)		
≥24	185	45	never	201	48.9
Disease duration (year)			1 ~ 3	61	14.8
<1	52	12.6	>3	149	36.3
1 ~ 3	166	40.3	Mental stress		
>3	193	47.0	YES	356	86.6
Education experience (year)			NO	55	13.4
<9	24	5.8	Acne		
9 ~ 12	48	11.7	YES	145	35.3
>12	339	82.5	NO	266	64.7
Fertility demand			Hairy		
YES	255	62	YES	225	54.7
NO	156	38	NO	186	45.3
Mother or sister has PCOS			Family history of psychological disorder		
YES	28	6.8	YES	26	5.8
NO	383	93.2	NO	385	94.2

### 3.2 Basic clinical feature of individuals with PCOS with depressive symptoms

Among the 411 participants, a total of 203 individuals exhibited symptoms of depression, resulting in a prevalence rate of 49.4%. Among these, 170 individuals (83.7%) experienced mild depressive symptoms, 31 (15.3%) had moderate symptoms, and 2 (1%) were classified as having severe symptoms. In the majority of cases, individuals primarily exhibited slight impairments in mood and social functioning. Specific clinical characteristics are outlined in Table 2 and Supplementary Table S1.

**Table 2:** Basic clinical feature of depression patients.

Variable	Total number of people	Percentage (%)
No depression	208	50.6
Depression	203	49.4
Mild	170	83.7
Moderate	31	15.3
Severe	2	1

### 3.3 Univariate analysis

The results of the univariate analysis indicate that there are statistically significant differences (*p* < 0.05) between the PCOS with depression group and individuals with PCOS without depression group in terms of disease duration, high-fat diet, frequent late-night activities, infrequent exercise, mental stress, acne, and a family history of mental disorders. The age, BMI, education duration, fertility requirements, family medical history of PCOS, and hirsutism showed no statistically significant differences between the PCOS with depression group and individuals with PCOS without depression group (*p* > 0.05; Table 3).

**Table 3:** Results of univariate analysis (*n* = 411).

Variable	No depression n(%)	Depression n(%)	*p*-value	Variable	No depression n(%)	Depression n(%)	*p*-value
Age			0.112	High-fat diet (>3x/week)			<0.001
<20	42(61.8)	26(38.2)		NO	132(59.7)	89(40.3)	
20 ~ 35	156(48.0)	169(52.0)		YES	76(34.5)	144(65.5)	
>35	10(55.6)	8(44.4)		Stay up late (>3x/week)			<0.001
BMI			0.294	NO	102(70.3)	43(29.7)	
<18.5	23(51.1)	21(48.9)		YES	106(39.8)	160(60.2)	
18.5 ~ 23.9	101(54.9)	83(45.1)		Frequency of exercise (week)			<0.001
≥24	86(47.0)	97(53.0)		never	57(39.6)	144(60.4)	
Disease duration (year)			<0.001	1 ~ 3	41(67.2)	20(32.8)	
<1	41(78.8)	11(21.2)		>3	110(73.8)	39(26.2)	
1 ~ 3	62(37.3)	104(62.7)		Mental stress			<0.001
>3	105(54.4)	88(45.6)		NO	46(82.1)	10(17.9)	
Education experience (year)			0.355	YES	162(45.6)	193(54.4)	
<9	12(50.0)	12(50.0)		Acne			
9 ~ 12	29(60.4)	19(39.6)		NO	149(56.0)	117(44.0)	0.003
>12	167(49.3)	172(50.7)		YES	59(40.7)	86(59.3)	
Fertility demand			0.831	Hairy			0.56
NO	80(51.3)	76(48.7)		NO	97(52.2)	89(47.8)	
YES	128(50.2)	127(49.8)		YES	111(49.3)	114(50.7)	
Mother or sister has PCOS			0.102	Family history of psychological disorder			0.009
NO	198(51.7)	185(48.3)		NO	20(76.9)	6(23.1)	
YES	10(55.6)	8(44.4)		YES	188(48.8)	197(51.2)	

### 3.4 Multivariate analysis

**Figure 1:**
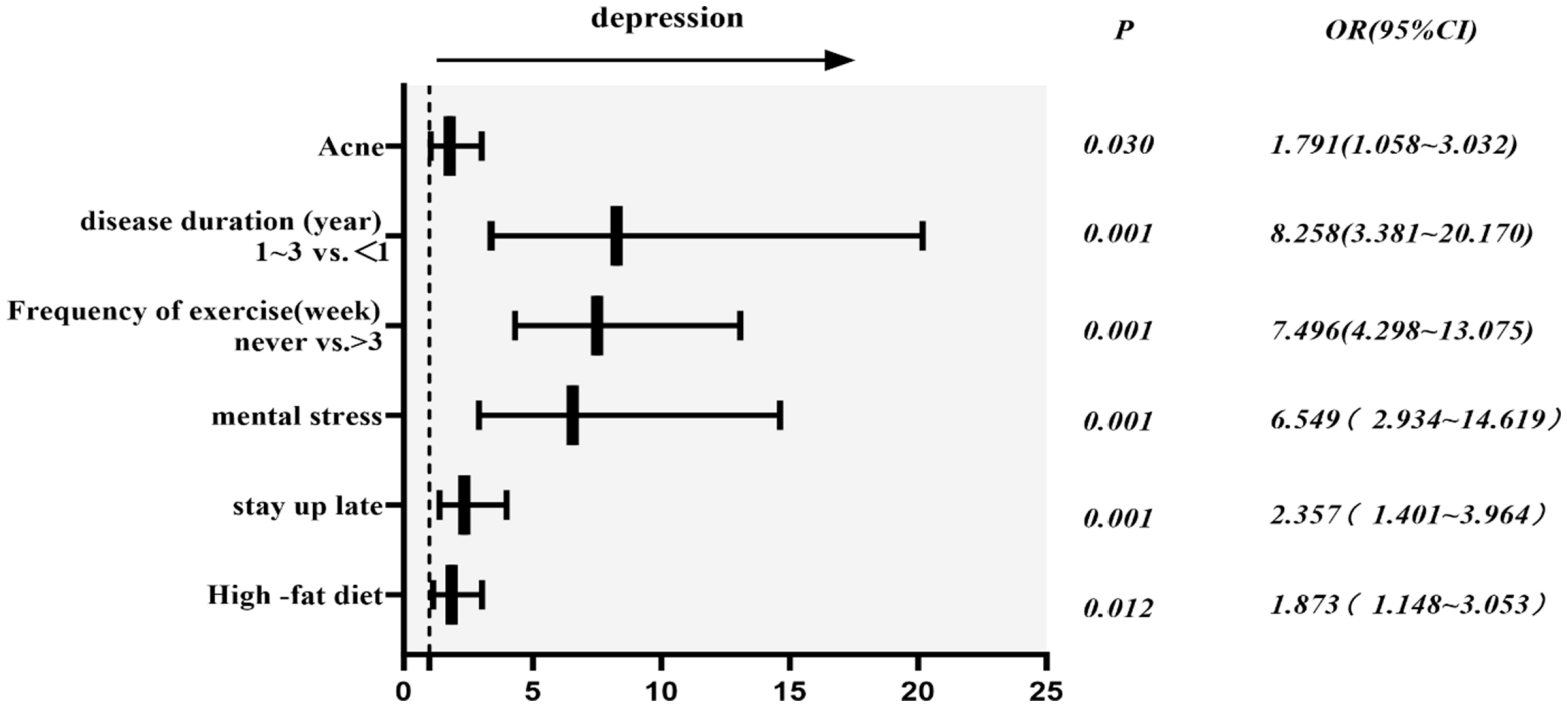
OR(95%CI) of risk factors.

Multivariate analysis revealed that high-fat diet, staying up late, mental stress, infrequent exercise, disease duration, and acne are all factors influencing the comorbidity of depression in individuals with PCOS. Frequent high-fat diet (frequent vs. infrequent: OR,1.873;95%CI,1.148 ~ 3.053), staying up late (frequent vs. infrequent:OR,2.357;95%CI,1.401 ~ 3.964), mental stress (yes vs. no: OR,6.549;95%CI,2.934 ~ 14.619), acne (present vs. absent: OR,1.791;95%CI,1.058 ~ 3.053), disease duration(1 ~ 3 year vs.<1 year: OR,8.258;95%CI,3.381 ~ 20.170), exercise (never vs. >3x/week: OR,7.496;95%CI,4.298 ~ 13.075). These factors are positively correlated with the incidence rate of comorbid depression in PCOS (Table 4; Figure 1).

**Table 4:** Results of multivariate analysis (*n* = 411).

	B	S.E.	Wald	*p*-value	OR	Lower limit	Upper limit
High-fat diet (>3x/week)	0.627	0.249	6.324	0.012	1.873	1.148	3.053
Stay up late (>3x/week)	0.857	0.265	10.447	0.001	2.357	1.401	3.964
Mental stress	1.879	0.410	21.038	0.001	6.549	2.934	14.619
Acne	0.583	0.269	4.709	0.03	1.791	1.058	3.032
Disease duration (year)							
1 ~ 3 vs.<1	2.111	0.456	21.470	0.001	8.258	3.381	20.170
>3vs.<1	0.810	0.432	3.518	0.061	2.248	0.964	5.240
Frequency of exercise (week)							
Never vs. > 3	2.014	0.284	50.377	0.001	7.496	4.298	13.075
1 ~ 3vs. >3	0.502	1.813	1.813	0.178	1.652	0.796	3.432

## 4 Discussion

PCOS is a prevalent reproductive endocrine disorder in women, primarily characterized by disturbances in reproductive function, metabolic irregularities, and psychological issues. In recent years, there has been significant research on reproductive and metabolic issues. However, the mechanisms underlying the comorbidity of depression in PCOS remain unclear. Additionally, the onset of depression in PCOS is often subtle, making it challenging to detect, and many individuals lack self-awareness (26).There is an issue of insufficient attention to the psychological and mental state of individuals with PCOS. Research indicates that the incidence of depression in individuals with PCOS ranges from 34 to 64%, and this rate shows an increasing trend over the years. Among them, reproductive-age women, particularly those between 20 and 35 years old, face a more significant risk of developing mental disorders (27). Reproductive-age women may experience a higher incidence of depression, possibly due to pressures related to childbirth, social and work-related stress. It is crucial to pay particular attention to the psychological well-being of this specific demographic.

In this study, the prevalence of depression in individuals with PCOS was found to be 49.4%, which is generally consistent with previous research. However, a noteworthy discovery in this study was that 83.7% of the individuals exhibited mild depressive symptoms, characterized by emotional distress without significant impact on social and occupational functioning. This suggested an increasing awareness of psychological issues in individuals with PCOS, highlighting the possibility of an overestimation of the incidence of clinical depression.

In our investigation, both univariate and multivariate analyses indicated a positive correlation between unhealthy lifestyle habits and the incidence of depression in individuals with PCOS. Individuals with PCOS were influenced by various factors such as menstrual irregularities, infertility, and hormonal disturbances. Most of them exhibit unhealthy lifestyle habits, including lack of physical activity, staying up late, high-fat diet, and mental stress (28), consistent with previous literature reports. The majority of individuals with PCOS often face barriers to physical activity. The reasons may include insufficient awareness, denial of the therapeutic benefits of exercise, poor compliance, lack of time, or a lack of confidence in maintaining a regular exercise routine (29). Clinical studies have found that a combination of exercise and medication can reduce patients’ weight, regulate female hormone levels, and decrease the severity of anxiety, depression, and other adverse emotional states (30). Additionally, both continuous and interval aerobic training have shown significant improvements in body image, anxiety, depression, and sexual dysfunction in individuals with PCOS (31). A recent meta-analysis (32) discovered a dose–response correlation between exercise and the likelihood of depression. Even participation in activities below the recommended threshold can yield substantial mental health advantages. Accumulating 2.5 h of brisk walking per week can decrease the risk of depression by 25% compared to those who are completely sedentary, while individuals engaging in half of this amount of activity can still lower their risk of depression by 18%. These connections can be elucidated by multiple mechanisms, such as the activation of acute neuroendocrine and inflammatory response pathways (such as the endocannabinoid system) during physical activity, as well as long-term adaptations, changes in neural brain structure, and factors like improved self-perception, body image, and increased social interaction. Animal experiments indicated that exercise enhances oxidative stress in PCOS rats, leading to changes in body composition and nutritional behavior (33).

Similarly, studies have found that poor sleep quality is positively correlated with depression scores in individuals with PCOS (34). The mental health status is closely related to sleep quality (35), and the occurrence of sleep disorders significantly increases in individuals with PCOS with depression (36). Sleep disorders are considered a risk factor for the onset and development of anxiety and depression (37). Sleep chronotype significantly influences sleep quality. Evening types, in comparison to morning or intermediate types, often experience a variety of sleep-related issues, including more frequent nightmares, shorter sleep duration, increased use of sleep medication, and poorer overall sleep quality (38). Late chronotype individuals, if they wish to adapt to their own biological rhythm, will experience some misalignment between their daily schedule and the natural light–dark cycle. Meanwhile, the natural light–dark cycle also influences human metabolic regulation. Compared to late chronotype individuals, those with an early chronotype exhibit a reduced risk of depressive symptoms, with an odds ratio of 0.79 (95%CI: 0.77–0.81), and a decreased risk of depressive disorders, with an odds ratio of 0.84 (95% CI: 0.82–0.88). Early chronotype individuals have a lower risk of depression and anxiety compared to late chronotype individuals, and they also experience lower disease severity when such conditions arise (39). Existing research offers substantial empirical evidence elucidating the link between chronotype and mental health (40). For example, a comprehensive population study involving 10,503 Finnish adults revealed a significant correlation between evening chronotype and heightened levels of depressive symptoms or clinical diagnosis of depression (41). In summary, these findings affirm that having an evening chronotype may indeed serve as a risk factor for the onset of depression. This is consistent with the results of our study. Therefore, the management of sleep issues should be considered as part of the overall care.

Cheng et al. (42) found through a lifestyle survey of individuals with PCOS that the main risk factors for emotional disorders include poor sleep quality, late bedtime, and high stress levels. The main protective factors include a light diet and regular exercise. Health management for individuals with PCOS should focus on aspects such as diet, exercise, and sleep. Lifestyle interventions significantly improve patients’ glucose and lipid metabolism, as well as their depressive and anxious conditions. They also contribute to the restoration of patients’ independent menstrual cycles (43). However, individuals with PCOS face the risk of disrupted eating attitudes and behaviors, which could impact the implementation of lifestyle interventions. Therefore, clinical practitioners should pay attention to the dietary habits of individuals with PCOS, not just focusing on weight loss outcomes (44). A study discovered (45) that a high-fat diet (HFD) not only induces anxiety and anhedonia but also disrupts intracellular cascades related to synaptic plasticity, insulin signaling/glucose homeostasis (including Akt, extracellular signal-regulated kinase (ERK), and P70 S6 K). Furthermore, it leads to heightened corticosterone levels and triggers activation of the innate immune system, resulting in elevated inflammatory cytokines (such as IL-6, IL-1β, and TNFα), thus impacting the onset of depression.

Some studies suggest that the increased risk of depression in individuals with PCOS may be related to the condition itself, such as weight gain, infertility, elevated androgens, insulin resistance, and high cortisol levels (46). While the severity of depressive symptoms increases with BMI in PCOS women, those with underweight PCOS also have a risk of developing depressive disorders. Other studies have found that the level of negative mood inhibition is unrelated to BMI (47). Wang (48) found that although the detection rate of depression in obese individuals with PCOS is higher than in non-obese patients, the difference is not statistically significant. Multiple studies (49–51) have also confirmed that there is no significant correlation between PCOS with depression and BMI. The results of our study indicated that factors such as fertility needs, hirsutism, and genetic factors are not associated with depressive symptoms. This aligns with the findings of Lin (49), who concluded that emotional disorders in PCOS are not significantly correlated with factors such as marital status, fertility, BMI, waist circumference, waist-to-hip ratio, hirsutism, acne, and acanthosis nigricans.

Additionally, mental stress is a significant risk factor for the development of depressive symptoms. While our research findings reveal a wide confidence interval in stress assessment using the Perceived Stress Scale, this could be attributed to significant variations in individual stress perception under similar circumstances. Moreover, our study solely relied on self-report methods, omitting objective indicators like heart rate and blood pressure, which might introduce inherent biases to the findings. However, existing literature extensively discusses the correlation between mental stress and depression in PCOS. Compared to individuals without PCOS, individuals with PCOS exhibit increased levels of depression, anxiety, and perceived stress. Stress may play a role in the association between PCOS, depression, and anxiety (52). The increase in stress affects female reproductive endocrine function, exacerbates metabolic disturbances and reproductive disorders, worsens the clinical phenotype of PCOS, and induces depressive emotions (53). Serum cortisol, as a potential stress marker, has been found to be increased in individuals with PCOS (54). Under stress, abnormal activation of the HPA axis leads to the release of glucocorticoids, triggering the activation of hypothalamic microglial cells that release inflammatory cytokines such as IL-1β and IL-6. This interference with hypothalamic neural signaling contributes to reproductive, metabolic disruptions, and emotional disturbances (55). Stress can elevate the migration of monocytes to the cerebrovascular system, particularly to the nucleus accumbens (NAc), a region associated with the brain’s reward center. These monocytes produce Matrix Metalloproteinase-8 (MMP-8), which plays a role in remodeling and regulating the extracellular matrix surrounding neurons in the brain. If MMP-8 leaks from the bloodstream into brain tissue, it can modify the matrix structure, consequently disrupting neuronal function. Mice subjected to this phenomenon display behavioral alterations similar to those observed in humans with depression (56).

In this study, we found that in addition to unhealthy lifestyle habits, acne is also a risk factor for depression in individuals with PCOS. Acne is a common clinical manifestation of hyperandrogenism in individuals with PCOS, which can affect women’s body image and potentially lead to psychological burden. Jiskoot (50) found that PCOS is associated with widespread psychological changes, which are related to acne (51). A cross-sectional study conducted in France (24), involving 24,452 participants, revealed that individuals with acne exhibited a greater frequency of depressive symptoms compared to those who had never experienced acne, which is consistent with the findings of this study.

Furthermore, our research revealed that a duration of illness of 1–3 years was also a risk factor for depression among individuals with PCOS. However, there is a scarcity of prospective longitudinal studies examining the mental health of individuals with PCOS. Despite the proven weakening of symptoms associated with certain PCOS characteristics, such as irregular menstruation, acne, and hirsutism, as age increases (57), a longitudinal, population-based study conducted within the Northern Finland Birth Cohort of 1966 revealed that the prevalence of depression remained unchanged among women reporting menstrual abnormalities and hirsutism at ages 31 and 46 (58). In a large, population-based, prospective cohort study involving black and white women, a 25-year follow-up observation revealed that women with PCOS experienced a heightened burden of depressive symptoms throughout their lifespan compared to their peers. However, the risk of depression gradually decreased as they aged (59). Although our study observed an elevated risk of depression among patients with a disease duration of 1–3 years, it is imperative to acknowledge the relatively wide confidence interval. Given the inherent limitations of a cross-sectional design, definitive evidence regarding the influence of medical history duration on depression status in PCOS patients is lacking. Consequently, the relationship between disease duration and depression status remains inconclusive, underscoring the need for additional research to provide more robust insights.

In conclusion, unhealthy lifestyles significantly influence the prevalence of depressive symptoms, and when multiple behaviors coexist, they often interact, exacerbating depressive symptoms (60). Our study further revealed that unhealthy lifestyles exert a significant influence on the depressive state of individuals with PCOS, underscoring the importance of lifestyle management for individuals with PCOS. This finding aligns with the recommendations outlined in the 2023 International Evidence-based Guideline for the Assessment and Management of Polycystic Ovary Syndrome (19). The guideline advocates for lifestyle interventions for all individuals diagnosed with PCOS, whether through exercise alone or a combination of diet, exercise, and behavioral strategies. These interventions aim to enhance metabolic health and address psychological concerns. Healthcare professionals should recognize that lifestyle management remains a central focus throughout the lifecycle of individuals with PCOS, emphasizing the importance of supporting healthy lifestyles.

## 5 Limitations and recommendations

This study investigated the degree of depression in individuals with PCOS and the influencing factors of related lifestyles. The findings highlight the significant prevalence of depressive symptoms in individuals with PCOS. Despite the higher incidence, most individuals experience mild depressive states, with slight impairments in work and social functioning. Early detection and intervention are crucial in preventing the progression of the condition. Depressive symptoms may impact the management and treatment adherence of PCOS. Therefore, efforts should be intensified to screen and address the mental health issues of individuals with PCOS (61). Women diagnosed with PCOS should not only receive standard medical care according to the guidelines but also comprehensive psychosocial and neurocognitive support to enhance their quality of life (62). However, this study has its limitations. Firstly, it is a cross-sectional study, which prevents making causal inferences. Secondly, this study is a single-center investigation predominantly focused on urban patients, limiting its generalizability. Future research should broaden the sample population to include individuals with PCOS from rural areas to provide sufficient information for the development of clinical intervention strategies. Thirdly, it is important to note that the sample size of this study is relatively small, and it solely concentrated on specific lifestyle factors such as high-fat diet, staying up late, and exercise habits. The study did not extensively investigate the specific patterns of each behavior in detail. For instance, concerning dietary habits, it solely focused on high-fat diet without taking into account the consumption of vegetables and fruits or regular eating patterns. Consequently, there might be some biases in the results. Fourthly, it is worth noting that the questionnaire employed a combination of interviews and self-reporting methods, and despite rigorous training, there remains a possibility of biases in the results. Fifthly, we utilized a stress perception assessment solely based on questionnaire forms, without incorporating physiological indicators like changes in blood pressure and heart rate, which could impact the generalizability of the study. However, this study also indicates that individuals with PCOS often experience mild depression, primarily characterized by depressive emotions and minor impairment in social and occupational functioning. Recognizing this can alleviate the psychological burden on patients, prevent unnecessary medical interventions, and prompt healthcare providers to focus on early psychological interventions for individuals with PCOS. Implementing effective measures to support patients in maintaining a healthy lifestyle is essential.

## 6 Conclusion

At a comprehensive hospital in China, the prevalence of depression among individuals with PCOS is 49.4%, with 83.7% exhibiting mild depressive symptoms. Unhealthy lifestyle factors emerged as the primary risk factors. These findings underscore the importance of heightened awareness among individuals with PCOS regarding the need for lifestyle adjustments. Furthermore, the majority of patients only manifest mild depressive emotions with slight impairment in work and social functions. This study alleviates the psychological burden on individuals with PCOS. Future research should delve into the potential causes of depression in individuals with PCOS and explore preventive measures to hinder the progression of depressive emotions into clinical depression.

## Data availability statement

The original contributions presented in the study are included in the article/Supplementary material, further inquiries can be directed to the corresponding author.

## Ethics statement

The studies involving humans were approved by the Ethics Review Committee of the First Affiliated Hospital of Henan University of Chinese Medicine (Approval No: 2023HL-200). The studies were conducted in accordance with the local legislation and institutional requirements. The participants provided their written informed consent to participate in this study.

## Author contributions

LL: Investigation, Methodology, Writing – original draft, Writing – review & editing. ZK: Resources, Writing – review & editing. PC: Resources, Supervision, Writing – review & editing. BN: Investigation, Writing – review & editing. YW: Data curation, Writing – review & editing. LY: Resources, Supervision, Writing – review & editing.
