# Supplementary material for: Association between mild depressive states in polycystic ovary syndrome and an unhealthy lifestyle
Source: Front Public Health. 2024 Apr 12;12:1361962. doi: 10.3389/fpubh.2024.1361962 (PMC11045954; doi:10.3389/fpubh.2024.1361962)
Supplement: Supplementary file 1 [file Table_1.DOCX]

Supplementary Material

Association between mild depressive states in polycystic ovary syndrome and an unhealthy lifestyle

**Lingling Li ^1,2,^ Zhiyuan Kang^1^, Ping Chen^1^,Baihan Niu^1^,Yaohui Wang^3^, Liping Yang^3*^ Correspondence:** Liping Yang, jc.ylping@hactcm.edu.cn.

# 1.Supplementary TableS1

| **TABLE S1 Basic clinical feature of depression patients.** | | | | | |
| --- | --- | --- | --- | --- | --- |
| Variable | Total number  of people | Percentage (%) | Variable | Total number  of people | Percentage (%) |
| no depression | 208 | 50.6 | **Agitation** |  |  |
| depression | 203 | 49.4 | 0.No abnormalities detected. | 116 | 57.1 |
| mild | 170 | 83.7 | 1.Some anxiety during the examination. | 69 | 34 |
| moderate | 31 | 15.3 | 2.Significant anxiety or frequent small movements. | 12 | 5.9 |
| severe | 2 | 1 | 3.Unable to sit still, stood up during the examination. | 1 | 0.5 |
| **Depression** | | | 4.Scratching hands, biting fingers, hair, and lips. | 5 | 2.5 |
| 0.Not appeared | 44 | 21.6 | **Mental anxiety.** | | |
| 1.Only complain when asked | 82 | 40.4 | 0.No abnormalities. | 51 | 25.1 |
| 2.Describe spontaneously during the interview | 47 | 23.2 | 1.When asked, they will talk about it. | 81 | 40 |
| 3.The emotion can be expressed through facial expressions, postures, voices, or the desire to cry without using words. | 26 | 12.8 | 2.Spontaneously express. | 36 | 17.3 |
| 4.The patient's self-expression and nonverbal expressions (facial expressions, actions) almost completely manifest this emotion. | 4 | 2 | 3.Expressions and speech reveal obvious anxiety. | 34 | 17.2 |
| **Guilt** |  |  | 4.Obvious panic. | 1 | 0.5 |
| 0. Not appeared | 88 | 43.3 | **Somatic anxiety.** | | |
| 1.Blame oneself and feel that one has already caused harm to others. | 56 | 27.6 | 0.Not appeared. | 40 | 19.7 |
| 2.Feel that one has committed a crime, or repeatedly think about past mistakes and faults. | 44 | 21.7 | 1.Mild. | 132 | 65 |
| 3.Feel that the illness is a punishment for one's mistakes or guilt, or have delusions of guilt. | 15 | 7.4 | 2.Moderate, with definite symptoms as described above | 31 | 15.3 |
| 4.Delusions of guilt accompanied by accusations or threatening fantasies. | 0 | 0 | 3.Severe, with severe symptoms as described above, affecting daily life or requiring treatment. | 0 | 0 |
| **Suicide** | | | 4.Seriously affecting daily life and activities. | 0 | 0 |
| 0. Not appeared | 149 | 73.4 | **Gastrointestinal symptoms**. | | |
| 1.Feel that life is meaningless. | 30 | 14.8 | 0.Not appeared. | 153 | 75.4 |
| 2.Wish that one had already died, or often think about matters related to death. | 16 | 7.9 | 1.Loss of appetite, but able to eat without encouragement. | 50 | 24.6 |
| 3.Negative thoughts (suicidal thoughts) | 8 | 3.9 | 2.Eating requires others to prompt or request, and the use of laxatives or digestive aids is necessary. | 0 | 0 |
| 4.Have severe suicidal behavior | 0 | 0 | **General symptoms** | | |
| **Difficulty falling asleep** | | | 0.Not appeared. | 79 | 38.9 |
| 0.No difficulty in falling asleep | 93 | 45.8 | 1.Heaviness in the limbs, back, or neck, back pain, headache, muscle pain, general fatigue or tiredness. | 124 | 61.1 |
| 1.Main complaint: Difficulty falling asleep, unable to sleep even after half an hour on the bed. | 95 | 46.8 | 2.Symptoms are evident. | 0 | 0 |
| 2.Main complaint: Difficulty falling asleep every night. | 15 | 7.4 | **Sexual symptoms** | | |
| **Shallow sleep** | | | 0.No abnormalities. | 21 | 10.3 |
| 0. Not appeared | 75 | 36.9 | 1.mild | 130 | 64.1 |
| 1.Shallow sleep with frequent nightmare | 109 | 53.7 | 2.Severe | 40 | 19.7 |
| 2.Woke up in the middle of the night. | 19 | 9.4 | Cannot be certain, or this item is not suitable for the evaluated person. | 12 | 5.9 |
| **Early awakening.** | | | **Hypochondriasis.** | | |
| 0. Not appeared | 86 | 42.4 | 0.Not appeared. | 87 | 42.9 |
| 1.There is early awakening, one hour earlier than usual, but able to fall asleep again. | 75 | 36.9 | 1.Excessive concern about the body: | 29 | 14.3 |
| 2.Early awakening with inability to fall asleep again. | 42 | 20.7 | 2.repeatedly considering health issues; | 80 | 39.4 |
| **Work and interest.** | | | 3.have a delusion of hypochondriasis and often seek medical attention due to concerns about illness. | 7 | 3.4 |
| 0. Not appeared | 51 | 25.1 | 4.delusion of hypochondriasis with hallucinations | 0 | 0 |
| 1.Only when asked will they talk about it. | 52 | 25.6 | **Weight loss** | | |
| 2.Spontaneously express a loss of interest in activities, work, or learning, directly or indirectly. | 97 | 47.8 | 0.Weight loss of less than 0.5kg within a week. | 144 | 70.9 |
| 3.The patient's work or entertainment in the hospital room lasts less than 3 hours. | 1 | 0.5 | 1.Weight loss of more than 0.5kg within a week | 30 | 14.8 |
| 4.Stop working due to illness. | 2 | 1 | 2.Weight loss of more than 1kg within a week | 29 | 14.3 |
| **laggardliness** | | | **self-knowledge** | | |
| 0.Normal thinking and language. | 167 | 82.3 | 0.Knowing one has a disease and manifesting as depression | 88 | 43.3 |
| 1.Mild retardation was found during the mental examination. | 35 | 17.2 | 1.Knowing one has a disease but blaming it on poor food, environmental issues, busy work, viral infection, or the need for rest. | 79 | 38.9 |
| 2.Significant retardation was found during the mental examination | 1 | 0.5 | 2.Completely denying the existence of a disease | 36 | 17.8 |
| 3.Mental examination is difficult to perform. | 0 | 0 |  |  |  |
| 4.Complete inability to answer questions (catatonia). | 0 | 0 |  |  |  |
